# A guide to using the Theoretical Domains Framework of behaviour change to investigate implementation problems

**DOI:** 10.1186/s13012-017-0605-9

**Published:** 2017-06-21

**Authors:** Lou Atkins, Jill Francis, Rafat Islam, Denise O’Connor, Andrea Patey, Noah Ivers, Robbie Foy, Eilidh M. Duncan, Heather Colquhoun, Jeremy M. Grimshaw, Rebecca Lawton, Susan Michie

**Affiliations:** 1 Centre for Behaviour Change, University College London, London, UK; 2 School of Health Sciences City, University of London, London, UK; 3 Clinical Epidemiology Program, Ottawa Hospital Research Institute, Ottawa, Canada; 4 School of Public Health and Preventative Medicine, Monash University, Melbourne, VIC Australia; 5 Women’s College Research Institute, Women’s College Hospital and Department of Family and Community Medicine, University of Toronto, Toronto, Canada; 6 Leeds Institute of Health Sciences, University of Leeds, Leeds, UK; 7 Health Services Research Unit, University of Aberdeen, Aberdeen, UK; 8 Department of Occupational Science and Occupational Therapy, University of Toronto, Toronto, Canada; 9 Department of Medicine, University of Ottawa, Ottawa, Canada; 10 Institute of Psychological Sciences, University of Leeds, Leeds, UK

**Keywords:** Theoretical Domains Framework, Guide, Methods

## Abstract

**Background:**

Implementing new practices requires changes in the behaviour of relevant actors, and this is facilitated by understanding of the determinants of current and desired behaviours. The Theoretical Domains Framework (TDF) was developed by a collaboration of behavioural scientists and implementation researchers who identified theories relevant to implementation and grouped constructs from these theories into domains. The collaboration aimed to provide a comprehensive, theory-informed approach to identify determinants of behaviour. The first version was published in 2005, and a subsequent version following a validation exercise was published in 2012. This guide offers practical guidance for those who wish to apply the TDF to assess implementation problems and support intervention design. It presents a brief rationale for using a theoretical approach to investigate and address implementation problems, summarises the TDF and its development, and describes how to apply the TDF to achieve implementation objectives. Examples from the implementation research literature are presented to illustrate relevant methods and practical considerations.

**Methods:**

Researchers from Canada, the UK and Australia attended a 3-day meeting in December 2012 to build an international collaboration among researchers and decision-makers interested in the advancing use of the TDF. The participants were experienced in using the TDF to assess implementation problems, design interventions, and/or understand change processes. This guide is an output of the meeting and also draws on the authors’ collective experience. Examples from the implementation research literature judged by authors to be representative of specific applications of the TDF are included in this guide.

**Results:**

We explain and illustrate methods, with a focus on qualitative approaches, for selecting and specifying target behaviours key to implementation, selecting the study design, deciding the sampling strategy, developing study materials, collecting and analysing data, and reporting findings of TDF-based studies. Areas for development include methods for triangulating data, e.g. from interviews, questionnaires and observation and methods for designing interventions based on TDF-based problem analysis.

**Conclusions:**

We offer this guide to the implementation community to assist in the application of the TDF to achieve implementation objectives. Benefits of using the TDF include the provision of a theoretical basis for implementation studies, good coverage of potential reasons for slow diffusion of evidence into practice and a method for progressing from theory-based investigation to intervention.

**Electronic supplementary material:**

The online version of this article (doi:10.1186/s13012-017-0605-9) contains supplementary material, which is available to authorized users.

## Background

Implementing new practices and/or changing existing practices in organisations, services and systems require changes in individual and collective behaviour. Changing behaviour requires an understanding of the influences on behaviour in the context in which they occur.

Behavioural theories provide an explicit statement of the structural and psychological processes hypothesised to regulate behaviour and behaviour change and are therefore relevant to investigating implementation problems and informing implementation interventions. There have been calls for more explicit use of theory to identify influences on behaviour change (i.e. facilitators of and barriers to change) [1, 2]; understand mechanisms of change, including how and in which contexts interventions are effective [3–5]; and inform implementation interventions [6–13]. Despite this, systematic reviews of interventions designed to change professional practice have shown only small numbers of rigorous evaluations reporting the use of theory to assess implementation problems or guide intervention design [8, 13, 14].

The Theoretical Domains Framework (TDF) was initially developed for implementation research to identify influences on health professional behaviour related to implementation of evidence-based recommendations and has been cited in over 800 peer-review publications (Web of Knowledge accessed April 2017). A synthesis of 33 theories of behaviour and behaviour change clustered into 14 (originally 12) domains [15, 16]; the TDF is a theoretical framework rather than a theory; it does not propose testable relationships between elements but provides a theoretical lens through which to view the cognitive, affective, social and environmental influences on behaviour.

In addition to understanding health professional behaviour, the TDF was extended to be relevant to other areas in which changing behaviour is important such as changing patient behaviours. Examples include increasing physical activity in children with motor impairments [17] and stroke survivors [18]. Other examples relate to changing general population behaviours, e.g. reducing loneliness in older adults [19] and increasing physical activity[20]. This article focuses on implementation.

Despite its extensive use in implementation research, no formal guidance exists on how to apply the TDF. In a study of using the TDF, health professionals from a range of disciplines reported that it increased their confidence in undertaking projects, provided a broad perspective and provided a means of understanding the implementation problem and potential solutions in theoretical terms. Reported challenges to using the TDF included lack of time and other resources and insufficient expertise to operationalise the TDF [21]. Participants suggested training and resources to support to use of the TDF. This guide is intended to address these challenges with the aim of making the TDF more useable by a wider audience of researchers and practitioners with an interest in implementation.

The guide begins by presenting a rationale for using behavioural theory to investigate and address implementation problems before describing the TDF, its development and main applications. The guide then describes the methodological considerations for using the TDF including selecting and specifying a target behaviour, selecting study design, deciding the sampling strategy, developing an interview schedule and collecting and analysing data. It aims to provide methodological and practical guidance to those interested in using the TDF to inform implementation efforts. We primarily focus on qualitative approaches (mainly interview studies) as this is the most common approach adopted when using TDF. Other potential approaches are discussed but in a lesser detail. Throughout the guide, implementation studies are presented to illustrate recommended methods and practical considerations. We finally discuss limitations, challenges and opportunities.

### Development of the TDF

Eighty-three theories of behaviour and behaviour change have recently been identified in a review across disciplines in social and behavioural sciences [22]. Selecting from such a large number of potentially relevant, sometimes overlapping, theories can be challenging. In an effort to make theories more accessible to those working in implementation, a team of behavioural scientists developed the TDF in collaboration with implementation researchers [15]. The TDF is an integrated theoretical framework synthesised from 128 theoretical constructs from 33 theories judged most relevant to implementation questions. The consensus process used by this cross-disciplinary group to develop the framework included (i) identifying theories and theoretical constructs relevant to behaviour change; (ii) simplifying these theories and constructs into overarching theoretical domains; (iii) evaluating the importance of the theoretical domains; (iv) conducting a cross-disciplinary evaluation and synthesis of the domains and constructs; (v) validating the domain list; and (vi) piloting a series of interview questions to elicit views about the constructs and domains. Whilst the domains cover the physical and social environment, the majority relate to individual motivation and capability factors. For clarity, the original version of the TDF is referred to in this guide as TDF(v1).

The TDF underwent a validation exercise with an independent group of behavioural experts to investigate the optimal structure and content of the framework [16]. The version of TDF, resulting from this validation, showed similar structure and content to the original with slight differences leading to 14 domains covering 84 theoretical constructs. For clarity, this 14-domain version of the TDF is referred to in this guide as TDF(v2). Both versions are used in research and practice, and given their similarity, either can be used depending on users’ familiarity and preference. Both versions are presented in Table 1.

**Table 1:** The Theoretical Domains Framework (v1 [15] and v2 [16]) with definitions and component constructs

Version 1[15]	
Domain	Constructs
Knowledge	Knowledge Knowledge about condition/scientific rationale Schemas + mindsets + illness representations Procedural knowledge
Skills	Skills Competence/ability/skill assessment Practice/skills development Interpersonal skills Coping strategies
Social/professional role and identity	Identity Professional identity/boundaries/role Group/social identity Social/group norms Alienation/organisational commitment
Beliefs about capabilities	Self-efficacy Control—of behaviour and material and Social environment Perceived competence Self-confidence/professional confidence Empowerment Self-esteem Perceived behavioural control Optimism/pessimism
Beliefs about consequences	Outcome expectancies Anticipated regret Appraisal/evaluation/review Consequents Attitudes Contingencies Reinforcement/punishment/consequences Incentives/rewards Beliefs Unrealistic optimism Salient events/sensitisation/critical incidents Characteristics of outcome expectancies—physical, social, emotional; sanctions/rewards, proximal/distal, valued/not valued, probable/improbable, salient/not salient, perceived risk/threat
Motivation and goals	Intention; stability of intention/certainty of intention Goals (autonomous, controlled) Goal target/setting Goal priority Intrinsic motivation Commitment Distal and proximal goals Transtheoretical model and stages of change
Memory, attention and decision processes	Memory Attention Attention control Decision-making
Environmental context and resources	Resources/material resources (availability and management) Environmental stressors Person × environment interaction Knowledge of task environment
Social influences	Social support Social/group norms Organisational development Leadership Team working Group conformity Organisational climate/culture Social pressure Power/hierarchy Professional boundaries/roles Management commitment Supervision Inter-group conflict Champions Social comparisons Identity; group/social identity Organisational commitment/alienation Feedback Conflict—competing demands, conflicting roles Change management Crew resource management Negotiation Social support: personal/professional/organisational, intra/interpersonal, society/community Social/group norms: subjective, descriptive, injunctive norms Learning and modelling
Emotion	Affect Stress Anticipated regret Fear Burn-out Cognitive overload/tiredness Threat Positive/negative affect Anxiety/depression
Behavioural regulation	Goal/target setting Implementation intention Action planning Self-monitoring Goal priority Generating alternatives Feedback Moderators of intention-behaviour gap Project management Barriers and facilitators
Nature of the behaviours	Routine/automatic/habit Breaking habit Direct experience/past behaviour Representation of tasks Stages of change model
Version 2	
Domain (definition)	Constructs
1. Knowledge (An awareness of the existence of something)	Knowledge (including knowledge of condition/scientific rationale) Procedural knowledge Knowledge of task environment
2. Skills (An ability or proficiency acquired through practice)	Skills Skills development Competence Ability Interpersonal skills Practice Skill assessment
3. Social/professional role and identity (A coherent set of behaviours and displayed personal qualities of an individual in a social or work setting)	Professional identity Professional role Social identity Identity Professional boundaries Professional confidence Group identity Leadership Organisational commitment
4. Beliefs about capabilities (Acceptance of the truth, reality or validity about an ability, talent or facility that a person can put to constructive use)	Self-confidence Perceived competence Self-efficacy Perceived behavioural control Beliefs Self-esteem Empowerment Professional confidence
5. Optimism (The confidence that things will happen for the best or that desired goals will be attained)	Optimism Pessimism Unrealistic optimism Identity
6. Beliefs about Consequences (Acceptance of the truth, reality, or validity about outcomes of a behaviour in a given situation)	Beliefs Outcome expectancies Characteristics of outcome expectancies Anticipated regret Consequents
7. Reinforcement (Increasing the probability of a response by arranging a dependent relationship, or contingency, between the response and a given stimulus)	Rewards (proximal/distal, valued/not valued, probable/improbable) Incentives Punishment Consequents Reinforcement Contingencies Sanctions
8. Intentions (A conscious decision to perform a behaviour or a resolve to act in a certain way)	Stability of intentions Stages of change model Transtheoretical model and stages of change
9. Goals (Mental representations of outcomes or end states that an individual wants to achieve)	Goals (distal/proximal) Goal priority Goal/target setting Goals (autonomous/controlled) Action planning Implementation intention
10. Memory, attention and decision processes (The ability to retain information, focus selectively on aspects of the environment and choose between two or more alternatives)	Memory Attention Attention control Decision making Cognitive overload/tiredness
11. Environmental context and resources (Any circumstance of a person’s situation or environment that discourages or encourages the development of skills and abilities, independence, social competence and adaptive behaviour)	Environmental stressors Resources/material resources Organisational culture/climate Salient events/critical incidents Person × environment interaction Barriers and facilitators
12. Social influences (Those interpersonal processes that can cause individuals to change their thoughts, feelings, or behaviours)	Social pressure Social norms Group conformity Social comparisons Group norms Social support Power Intergroup conflict Alienation Group identity Modelling
13. Emotion (A complex reaction pattern, involving experiential, behavioural, and physiological elements, by which the individual attempts to deal with a personally significant matter or event)	Fear Anxiety Affect Stress Depression Positive/negative affect Burn-out
14. Behavioural regulation (Anything aimed at managing or changing objectively observed or measured actions)	Self-monitoring Breaking habit Action planning

### Use of the TDF in published implementation research

The TDF has been applied across a wide range of healthcare settings and clinical behaviours and was the subject of a thematic series appearing in *Implementation Science* (http://www.implementationscience.com/series/TDF) [23]. It has been the explicit basis of studies with a range of objectives and designs including the following:

- Identifying influences on behaviours. Exploration of barriers and facilitators to implementing specific evidence-based behaviours. Examples of interview studies include investigating facilitators and barriers to offering a family intervention to families of people with schizophrenia [24], transfusing with red blood cells [25, 26], discussing human papillomavirus (HPV) vaccination with patients [27], routinely ordering pre-operative tests [28], error-free prescribing [29], managing acute low back pain without ordering an X-ray [30], dementia diagnosis and management [31] and mild traumatic brain injury management [32]. Examples of questionnaire studies include investigating facilitators and barriers to hand hygiene [33], providing tobacco use prevention and cessation counselling among dental providers [34] and midwives engaging with pregnant women to stop smoking [35].
- Systematic intervention design. Examples include GPs, physiotherapist and chiropractors to manage acute low back pain [36, 37]; emergency department staff management of mild traumatic brain injury [38]; hospital clinician adherence to national guidelines on the management of suspected viral encephalitis [39]; and implementation of guidelines to promote safe use of nasogastric tubes [40].
- Process evaluations of randomised trials to better understand the effect of implementing evidence, e.g. in the Canadian CT Head Rule trials among emergency physicians [41].
- Guidance on identifying behaviour change techniques [7, 42] and designing broader intervention strategies [43].

The TDF has also been used beyond health, for example, to behaviours related to recycling behaviours [44]. References for published example applications of the TDF are presented in Additional file 1.

## Methods

Researchers from Canada, the UK and Australia representing health psychology, sociology, implementation and health services research, statistics and a range of clinical disciplines, including general practice, occupational therapy and chiropractic, participated in a 3-day collaborative meeting in December 2012 to discuss the state of the science in using the TDF in implementation research and to identify areas needing further development to advance its application. Specific objectives of the meeting were to:

- Review current evidence for TDF
- Identify gaps in the evidence and develop a plan to build an international collaboration among researchers and decision-makers interested in advancing the use of TDF
- Outline an agenda for a series of studies focused on the TDF

Participants had experience of using the TDF to assess implementation problems, design interventions and/or understand mechanisms of change. In reviewing gaps in TDF research and drawing on their collective experience using the TDF, the group identified that a guide to using the TDF would be useful to those applying it in implementation research.

To produce this guide, the group identified key steps in applying the TDF from selecting a behaviour to change through analysing and reporting data. The group’s experience and expertise were pooled to elaborate each of these steps. The group selected examples from the literature that best illustrated each of these steps to provide readers with instruction on how to use the TDF and examples of applications.

## Results

This section describes a range of methods used in TDF-based implementation research but guidance focuses on qualitative approaches—primarily interviews and focus groups. Stages for conducting TDF-based research are presented in Table 2.

**Table 2:** Stages in conducting TDF-based implementation research

Stage	Detail	Key considerations
1. Select and specify the target behaviour/s	Use documentary analysis or empirical research to identify and specify who should do what differently, to increase the uptake of evidence-based practice	May require assessment of the feasibility of measuring the behaviour as an outcome variable
2. Select the study design	May involve semi-structured individual interviews, focus group interviews, questionnaires, structured observations, documentary analysis or consensus processes	Design should fit the research question and will depend on the stage of investigation through exploration and development to intervention and explanation
3. Develop study materials	Although materials from previous studies may be used as templates, materials should be adapted to be appropriate to the specified behaviour/s and context	Requires in-depth understanding of the theoretical content of each domain Requires pilot testing for comprehensibility and clinical sensibility
4. Decide the sampling strategy	For exploratory studies, a maximum variation approach is appropriate	Key participants are those who will, or should, perform the target behaviour but other stakeholders (e.g. managers, co-workers) may also contribute a valuable perspective
5. Collect the data	Published studies have used audio-recorded interviews (face-to-face or telephone; one-to-one or focus group) or questionnaires (paper-based or online)	Effective interviewing requires standard interviewer competencies and in-depth understanding of the theoretical content of each domain
6. Analyse the data	The objective is to identify the domains that are most relevant to the implementation problem being addressed and to populate those domains with context-relevant and behaviourally specific content	Coding in qualitative studies requires in-depth understanding of the theoretical content of each domain
7. Report findings	For interview studies, report presents tables that include illustrative quotations, specific beliefs identified (with frequencies, if appropriate) and classification into domains	The explanatory text relating to the table of course relates to the study objectives

The steps are selecting and specifying a target behaviour, selecting study design, deciding the sampling strategy, developing an interview schedule and collecting and analysing data. Each step is described in detail and accompanied by an example from the published implementation research literature.

**Fig. 1:**
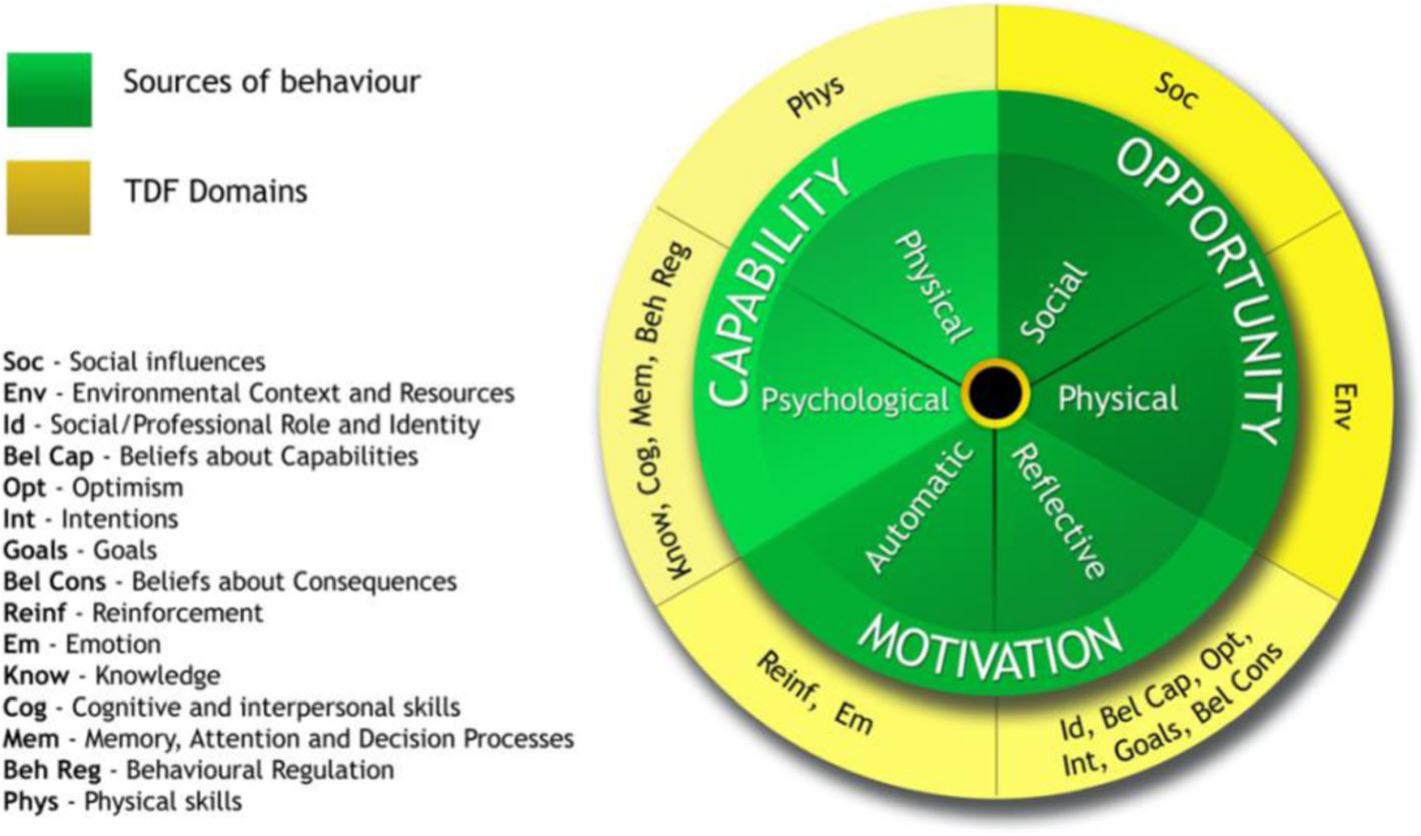
Flow chart illustrating steps to analyse interview transcripts to select a theoretical basis for designing a questionnaire study

1. Select and specify the target behaviour/s
  The first step is to identify the behaviour(s) that need to be changed to address the implementation problem. In the contexts in which they are performed, the key behaviours are often interdependent with other behaviours within the individual and with behaviours of others. Other attributes include the inherent complexity of behaviour, including whether it is performed by individual healthcare professionals or by healthcare teams, and the frequency of opportunities for performing the behaviour.
  The next step is to specify these behaviours in terms of who needs to do what differently, when, where, how and with whom? If there are multiple behaviours, it is helpful to start with one or, possibly, two behaviours to target in the first instance. The criteria to consider when prioritising behaviours include (i) how modifiable it is likely to be and (ii) how central it is in bringing about the desired change in clinical practice; (iii) the ‘spillover’ effect, i.e. the positive or negative effect on other related behaviours if change occurred (known in the literature as conflicting and facilitating behaviours) [45]; and (iv) the amenability to measurement. Selection is usually influenced by a thorough assessment based on a range of sources of information about the problem and careful examination of evidence-based recommendations and empirical research, both published and local. There are inevitable trade-offs in prioritising behaviours for investigation.
  The more precisely the behaviour is specified, the greater the specificity of the facilitators and barriers identified. There are three aspects of this process: (1) decide the appropriate level of behavioural specificity; (2) identify who performs the behaviour, when, where and how; and (3) consider the attributes of the target behaviour such as complexity, action sequences and interdependence of team-level behaviours. We explain each of these aspects below, using examples from the implementation research literature to illustrate each point.
  Behavioural specificity
  There is a balance between being highly behaviourally specific (to maximise the likelihood of identifying barriers to and facilitators of that behaviour) and being general enough to be relevant to a range of contexts. For example, to investigate the management of diabetes in primary care, a more specific description is ‘general practitioners measure the blood glucose levels of their patients with diabetes every 6 months’ whereas a less specific description is ‘general practitioners managing their patients with diabetes according to guidelines’. The more specific description is more likely to identify the sources of implementation problems that need to be changed because it is clear what the behaviour is, who needs to perform the behaviour and how often it is performed. Thus, study findings are more likely to be interpretable if the behaviour targeted for change is defined carefully in terms of *who* needs to perform the behaviour, *what* they need to do, *when* they need to do it, *where* they need to do it, *how often* they need to do it and *with whom* will they need to do it [43, 46, 47]. Furthermore, it is important that the behaviour be specified in terms of *target* behaviour, e.g. GPs to advise patients with sore throats to take painkillers and drink plenty of cool or warm fluids, rather than the *problem* behaviour, e.g. GPs prescribing antibiotics for sore throats. There are cases where it is not possible to isolate and target one behaviour for change, for example if designing an intervention to help GPs improve diabetes control; there are more than 10 interdependent behaviours that could be targeted for change. One way of addressing this challenge is to prioritise two or three key behaviours. The example in Table 3 illustrates the specification of a professional behaviour according to the principle of behavioural specificity. It may be that a goal has been set, e.g. reducing infections in a particular setting, but the behaviours required to achieve that goal are not immediately obvious. In these cases, analysis of audit data and discussion with stakeholders can support the identification of relevant behaviours and agreement on target behaviours.
2. Select the study design
  As with all research, the appropriate study design depends on the research question and the state of knowledge in the given field of research. For example, qualitative interviews may be more useful when little is known about an implementation problem and the study design allows researchers to probe in greater detail providing richer data which can be helpful when developing theory-informed interventions in that they may provide better insight into needed content of interventions. They are also likely to be useful to understand the mechanism of action in interventions. Survey studies may be more relevant when more is known about the problem and potentially relevant influencing factors, but the aim is to identify those factors/domains predictive of behaviour change in a more representative sample, or to explore mechanism of action of interventions (mediation analyses). Structured observation and approaches such as documentary analysis may be useful to supplement interview/survey studies (data can be triangulated), but they are unlikely to be sufficiently comprehensive for all domains (for example cognitions are not observable or documented). As the TDF has largely been applied at exploratory and formative stages of a research programme to inform problem analysis and intervention development, most reported work have used qualitative interviews (one-on-one or focus groups) to elicit health professionals’ perceptions of TDF-related barriers and facilitators. However, the TDF is potentially applicable to other research designs for which methods can be further developed, e.g. structured observations, documentary analysis, case study designs.
  The TDF has been used in questionnaire studies (Table 4). There are three published validated questionnaire measures of the TDF to identify influences on the following behaviours: health care professionals’ patient safety behaviours [48], physical activity in the general population [20] and generic health professional behaviours [49].
  The TDF also has the potential to inform systematic reviews by synthesising influences on behaviours across studies according to theoretical domains (Table 5) and understanding effect size (Table 6).
3. Decide the sampling strategy
  The target population needs to include the target adopters of the behaviours and/or other relevant stakeholders. These could be individuals (e.g. clinicians, patients, students or members of the public), dyads (e.g. clinicians and patients; teachers and students) or teams (e.g. teams of clinicians and managers or business workgroups). The organisational level at which change is proposed to occur could be at different levels, e.g. individual, team, organisation or population levels. Change may need to be coordinated across different organisational levels [50], with different types of behaviours being enacted by a range of individuals or groups.
  There are several challenges to collecting data for implementation research which need to be considered when deciding sampling strategy. First, studies have largely relied on self-report data and individuals may be biassed in their views about the problem and attribute failures to external (environment or other people) rather than internal (ability, effort) factors [51, 52]. Therefore, it is important to include multiple perspectives (e.g. from users, managers, commissioners as well as providers of health care) and, where possible, to use multiple sources of data (e.g. clinician self-report of influences on behaviour via interviews and/or surveys, practice and policy documents and direct observation of behaviour) [53, 54]. In this way, the validity of findings are likely to be improved through integration or ‘triangulation’. Triangulation is the ‘process of studying a problem using different methods to gain a more complete picture’ [55]. A number of triangulation techniques are available to researchers, and integration can be carried out at the analysis and/or interpretation stages (for an overview of methods, see O’Cathain et al. [55]).
  Although sample size for interview and focus group studies can be determined by the sampling procedure (such as purposive sampling for maximum variation) and the implementation problem under investigation, specifying a minimum sample size a priori is recommended. Francis et al. recommend that a minimum of 10 interviews be conducted for initial data analysis, followed by three additional interviews until no new theme emerges (stopping criterion) [56]. Sample size will also depend on whether the study involves different groups of health care professionals and whether they are being analysed together or as separate groups. If multiple groups are involved and the plan is to analyse them separately to get varying group perspectives, 10 interviews plus three per group are advised as a minimum (Francis et al. [56]). Table 7 illustrates the selection of professional stakeholder groups to achieve maximum variation in the sample of a TDF-based qualitative interview study. Focus groups involving all stakeholders have the potential to provide multiple perspectives and potentially reduce the tendency to focus only on external influences on behaviour. Our recommendations would be a minimum of 3 groups if the focus is on a specific care setting.
4. Develop interview schedule
  Interview schedule
  As in all interview studies, a key step in a TDF-based interview study is the development of an interview schedule. We advise using language relevant to the target population and piloting schedules to check comprehension. The schedule typically consists of an open question for each theoretical domain to elicit the first response, followed by a series of follow-up prompts to probe more deeply. Each question focuses explicitly on the target behaviour. The TDF was developed to promote a comprehensive consideration of possible influences on a given behaviour so there is no specific order in which the questions should be asked. We recommend flexibility in the order in which domains are covered to harness the natural flow of the conversation if a respondent volunteers’ information relating to a domain not yet covered. The number of domains covered and number of questions within each domain depend on the target behaviour and existing evidence. For example, where previous research has established a domain is not relevant to a target behaviour, researchers may consider omitting questions relating to that domain and focusing more on exploring domains considered more relevant to the target behaviour. However, as with all qualitative research, coding using the TDF can only code the text in the interview transcript. If questions are not asked, the text cannot be coded. Researchers will have to determine the value of including all domains to ensure coverage whilst balancing the evidence surrounding the target behaviour. An in-depth understanding of the theoretical content of domains and context of the implementation problem will help ensure interview schedules elicit a maximum amount of useful information. Published TDF questionnaires may be helpful in developing interview schedules [20, 48, 49]. Example questions to explore domains in implementation research taken from Huijg et al. [49] are provided in Additional file 2.
5. Collect the data
  Data can be collected using the TDF by one or more of the following: interviews, surveys, observation and documentary analysis. For example, in a study to inform implementation of a hospital care pathway to reduce sepsis mortality, the TDF was used to gather data by interviews with the care team, observation of ward staff and analysis of hospital protocols. Behaviour change techniques identified through observation and interview were then mapped to TDF to identify mechanisms of action [54]. Interviews can be conducted in groups or individually either face-to-face or by telephone. Our experience has been that interviews typically last on average between 25–45 min for one-on-one interviews and 50–90 min for focus group interview but is of course dependent on the number of behaviours being investigated.
  As with all interviewing, follow-up questions are the key to eliciting a good understanding of the ways in which the domains contribute to the target problem or could be used to bring about change. For example, a question such as ‘how confident are you in doing x?’ with a follow-up probing question ‘what has made you confident?’ allows for the participant to be specific as they reflect on their confidence but also gives them an opportunity to reflect on situations that limit their confidence. Anchoring discussion to the target behaviour(s) can help to keep discussion focused and avoid a drift into general issues. As with developing study materials, interviewers with an underlying understanding of the theoretical constructs underpinning domains will promote appropriate probing during interview.
6. Analyse the data
  The TDF is intended for use by researchers and practitioners from many disciplines. Whilst users do not necessarily need expertise in using particular theories, a good understanding of the domains and the theoretical constructs each represents are recommended to aid interpretation of data. Data can be analysed deductively, using the TDF to generate the framework for a content analysis and, inductively, generating themes that can then be considered in relation to domains. Some research teams have used this approach as the basis for designing predictive questionnaires to collect quantitative data to test out hypotheses generated by the qualitative analysis (Fig. 1). Intervention designers can select behaviour change techniques either directly from identified relevant theoretical domains using validated linkages [7, 42] or by linking to the Behaviour Change Wheel to guide the selection of intervention functions, policy categories and behaviour change techniques [43].
  Develop a coding guideline
  A coding guideline is a set of explicit statements of how the TDF is to be applied to a specific data set. Statements provide guidance on strength of confidence that a piece of text indicates a domain where change is likely to be helpful in changing behaviour.
  The coding guideline should be developed at the same time as the interview schedule and updated iteratively during data collection. Independent coding by two people allows discrepancies to be discussed and coding guidelines refined until acceptable reliability is achieved between coders. Coding difficulties may arise because the interviewer has not sufficiently probed to clarify how responses relate to the domain under investigation. Reviewing transcripts during the data collection period rather than at the end will allow interviewers to refine the interview schedule as more understanding of the problem being studied is gained. Many uncertainties by the coding team can be eliminated if the interviewer is familiar with the implementation problem under investigation, the TDF and published studies using it.
  Deductive analyses
  Coding interview transcripts into theoretical domains: Coding begins by reading participants’ responses in the transcript, considering their relevance to the definitions of the domains and/or the constructs within the domains and then attributing them to one or more domains. This directed content analysis technique is guided by theory and/or relevant research findings to interpret meaning from the content of qualitative data for initial codes [57]. New users are advised to ensure that all coded texts relate to the target behaviour and not other behaviours interviewees or focus group participants may discuss but which are not relevant. Whilst the domains are purposively design to be broad groupings of the possible factors to influence behaviour, the intent is to explore the important domains in further detail. Some text may seem to fit in multiple domains. For example, everything a HCP does can be dependent on context so everything can be coded in environmental context and resources. However, this is a somewhat simplistic assumption and other domains permit the division of the contextual factors influencing behaviour and identify those that are amenable to change (i.e social context reflected in social influencing, reinforcement likely delivered by the context, beliefs about capabilities which are typically situation-specific and organisations which can make certain actions easier or more difficult). Text should be coded into the domains that best reflect the key theme, despite the inclination to code everything into one domain. Users with no or limited experience with the TDF should initially meet frequently to discuss coding and challenges to address concerns early in the process. Once the users are comfortable with the TDF, it is recommended that two researchers code data independently into theoretical domains following a mutually agreed coding guideline to increase the reliability of coding.
  Disagreements in coding are not uncommon, and discussions to resolve them can be informative both about the process and the substantive questions addressed by the study. In order to facilitate consensus among coders, we recommend that the coders articulate their understanding of the coded text (i.e. key meaning) and justify their rationale for selecting the domain. Justification for why the text should not be coded in the alternate domain should also be discussed, with each coder given the opportunity to discuss the other’s point. When consensus cannot be reached, discussion with an expert in the area being studied and an experienced researcher who is familiar with the TDF and the theories from which it draws can help guide coders to interpret the text in relation to the domains and the theoretical constructs within the domains. When agreement on assigning text to a single domain cannot be reached, consensus can be achieved by assigning the text to all the domains identified by both the coders. Coders should document which text is attributed to which theoretical domain/s. Table 8 illustrates this step.
  Data saturation: Data saturation is reached when the data collected do not contribute any new information about barriers and facilitators influencing the implementation problem. If data are collected progressively, concurrent with analysis, this will inform final sample size [58]. A number of factors can influence saturation including the design or scope of the study [59], the heterogeneity of the population [60] and the nature of the implementation problem [61].
  Reliability: Reliability between the two coders may be assessed by an inter-rater reliability coefficient, for example through assessing a kappa score across all domains [28]. Calculating simple percentage agreement can also be used to establish agreement among coders [62]. Reliability between two coders is acceptable if kappa score > 0.6 or percentage agreement > 60% is achieved [63]. PABAK kappa which corrects for negative agreement when Cohen’s kappa is marginal can also be used.
  Statistical software: NVivo software, or other qualitative analysis package, may be used for data analysis. It can enhance analysts’ efficiency at data storage, retrieval, coding, editing and revising coding, organising data and sharing files across researchers and can be used to assess reliability.
  Inductive Analyses
  Generating themes and/or belief statements: For thematic analyses, researchers are encouraged to follow established methods [64, 65] and report explicitly the processes used to analyse the data.
  After coding data into theoretical domains, some researchers have used the following methods to further analyse the data within domains: generate overarching themes for a number of responses with similar underlying ideas and/or generate statements of specific underlying beliefs for each response [26]. The overarching themes represent the factors which are perceived to influence performance of the target behaviour. A belief statement is a collection of responses with a similar underlying belief that suggest a problem and/or influence of the beliefs on the target implementation problem [25]. For example, these responses, ‘guidelines are just guidelines’, ‘guidelines are not gospel’ and ‘there are no rules about going outside guideline’, were grouped under the belief statement ‘I can make my decision outside the guidelines’ [26]. For efficient use of time, one coder can generate belief statements and the other coder can interrogate and confirm those. In the example given above reported in Francis et al. [15], this step resulted in a list of belief statements supported by responses made in the interviews within each theoretical domain. Each belief statement was counted once within each interview to generate a frequency count across all interviews. This step in the analysis results in a list of belief statements with frequency counts for each of the belief statements and/or overarching themes within each theoretical domain. Frequency count of belief statements is not warranted in the case of focus group interviews as nonverbal behaviour such as nodding in agreement with a belief statement from another participant would not always be captured. Also, social influence effects may elicit more agreement with such a statement than would be identified in one-to-one interviews.
  Challenges in coding data
  Sometimes there can be uncertainties when coding data into theoretical domains, a feature not uncommon in qualitative coding. This should not impede progress of the study. Coding into theoretical domains may require a certain amount of interpretation of theoretical constructs by researchers.
  Coding interview transcripts into some theoretical domains can be more challenging compared to other domains depending upon the implementation problems under investigation. A single response may involve more than one theoretical domain, and often, several domains are addressed in a single response to a particular interview question (see Additional file 3 for examples). An attempt to tease out different domains for the purpose of coding into appropriate theoretical domain may result in losing the context of the response. To avoid such occurrences, we recommend that the entire response be coded in all identified domains.
  Sometimes responses may not clearly fit any theoretical domain despite guidance on interpretation by trained psychologists or those with a good understanding of the TDF from the study team. It is important to note such occurrences as these effectively test the capacity of the TDF to account for all the interview data. The most common reason for utterances not fitting into a domain is that it is not about the target behaviour. Coding difficulties then become an opportunity to check the adequacy of the behavioural specification. Hence, we recommend coding wider contextual information into a separate code for ease of retrieval and completion of descriptive summaries of participants and their practice characteristics.
  Identification of relevant theoretical domains: In this step, relevant theoretical construct domains (i.e. domains that should be targeted in an intervention) are identified by judging the importance of specific beliefs or themes. The following three criteria have been applied in published studies: (1) relatively high frequency of specific beliefs and/or themes (not relevant to focus group interviews); (2) presence of conflicting beliefs; and (3) evidence of strong beliefs that may affect the target behaviour [28]. At the completion of this step, the researchers will have a list of relevant theoretical domains that are most likely to influence the target implementation problem and associated behaviours. Table 9 illustrates this point.

**Table 3:** Specification of the target behaviour according to the principle of behavioural specificity

Study title
Evaluation of a TDF-informed implementation intervention for the management of acute low back pain in general medical practice
Rationale for changing behaviour
Management of low back pain in general medical practice is common, but this management is not always concordant with recommended evidence-based guidelines. In particular, x-rays are overused which leads to unnecessary harm due to radiation exposure and possible detection of incidental irrelevant findings, and an intervention of known effectiveness, giving advice to stay active, is underused.
Study design and materials
Three phase study: 1. Qualitative methods: focus groups with general practitioners (GPs) (*n* = 42) using TDF to identify barriers to and facilitators of two evidence-based target behaviours related to the management of acute low back pain: one related to diagnosis, that plain film x-rays are necessary only if fracture is suspected, and one related to treatment, that of providing advice to stay active, including the avoidance of advising more than two days of bed rest. Here is an example of specifying these behaviours using the criteria: *Who* is performing the behaviour? *What* do they need to do? *When* do they need to do it? *Where* do they need to do it? If applicable, the behaviour should also be specified in terms of *how often* and *with whom* it should be done. *Behaviour 1: Manage patients without referring for plain X-ray* *Who*–GPs *What*–Manage patients with acute low back pain without referring for plain X-ray *When–*On assessment or review of patients presenting with acute, uncomplicated low back pain of less than 3 months duration and without any serious underlying pathology suspected *Where*–In clinic *How often*–On assessment and review *With whom*–Behaviour not depended on others *Behaviour 2: Provide advice to stay active* *Who*–GPs *What*–Provide advice to stay active *When–*When managing patients with acute, uncomplicated low back pain of less than 3 months duration and without any serious underlying pathology suspected *Where*–In clinic *How often*–On assessment and review *With whom*–Behaviour not depended on others 2. Intervention development: mapping of barriers and facilitators within TDF domains to behaviour change techniques (detail provided in French et al. [36]). The TDF was used to guide the choice of behaviour change techniques and intervention components. 3. Cluster randomised trial: evaluation of a TDF-based intervention compared to simple dissemination of the guideline (results provided in French et al. [69]). Outcomes measured included behavioural predictors (e.g. knowledge, attitudes and intentions), fear avoidance beliefs, behavioural simulation (clinical decision about vignettes) and rates of X-ray and CT-scan (medical administrative data). Forty seven practices (53 GPs) were randomised to the control and 45 practices (59 GPs) to the intervention.
Findings and conclusions
The TDF allowed for the systematic identification of multiple barriers and facilitators in general medical practice and subsequent mapping to behaviour change techniques. The intervention consisted of interactive workshops designed to improve the knowledge, skills, intentions and clinical decision-making of the general practitioners. The intervention had some influence on GP adherence to an evidence-based guideline for the management of lower back pain at 12 months post-intervention. Overall, the intervention led to small changes in GP intention to practice in a manner consistent with an evidence-based guideline, but it did not result in statistically significant changes in actual behaviour measured via administrative data.
Study outputs
French et al. [36, 69]; Page et al. [70]; McKenzie et al. [71]

**Table 4:** Using a TDF questionnaire to understand an implementation problem; the example of designing hospital patient safety interventions

Study title
The demonstration of a theory-based approach to the design of localized patient safety interventions
Rationale for changing behaviour
Between 3.7 and 17.7% of patients in hospital are inadvertently harmed either by healthcare professional error or deviations from recommended practice. In this example, the TDF was used to understand behaviours related to implementing a patient safety guideline promoting safe nasogastric feeding.
Study design and materials
The Influences on Patient Safety Behaviours Questionnaire IPSBQ [48], a 34-item tool based on the 12-domain version of the TDF was completed by staff in three hospitals to identify influences on locally identified target behaviours relating to safe nasogastric feeding. MANOVA was used to identify highest scoring domains.
Findings and conclusions
Social influences, environmental context and resources, skills and emotion were identified as the most influential domains. Relevant domains were further explored in focus groups and intervention strategies generated using explicit links between theoretical domains and behaviour change techniques [7].
Study outputs
Taylor et al. [72]

**Table 5:** Using the TDF to synthesise evidence; the example of barriers to diabetes management in primary care

Study title
Identifying barriers to primary care type 2 diabetes management: qualitative systematic review
Rationale for changing behaviour
There is broad consensus and a strong evidence base to guide the care of diabetes. Despite encouraging trends in the delivery and outcomes of care for people with diabetes, there remains significant scope for improvement. Most clinical management of diabetes now occurs in primary care. Interventions to enhance the implementation of evidence-based guidelines to improve the care of people with diabetes have shown small to modest effects. To ensure that interventions address barriers to behaviour change and build on known facilitators, it is important to understand primary care clinicians’ beliefs around their day-to-day management of such patients.
Study design and materials
Systematic review of qualitative studies, including searches of following databases from 1980 to 2013: MEDLINE, EMBASE, CINAHL, PsycINFO and ASSIA. Qualitative studies examining diabetes management in primary care were eligible. Following screening of abstracts and full texts, data were coded to TDF domains and other themes if required. This review focused on behaviours to address clinical targets (including control of blood sugar, cholesterol and blood pressure) and processes of care (including foot examination). Findings were synthesised to identify barriers and facilitators common across or unique to clinical management goals, as well as apparent and potentially unexplored gaps in the literature.
Findings and conclusions
Out of 32 included studies; 17 address general diabetes care, 11 glycaemic control, three blood pressure, and one cholesterol control. Clinicians struggle to meet evolving treatment targets within limited time and resources and are frustrated with resulting compromises. They lack confidence in knowledge of guidelines and skills, notably initiating insulin and facilitating patient behaviour change. Changing professional boundaries have resulted in uncertainty about where clinical responsibility resides. Accounts are often couched in emotional terms, especially frustrations over patient adherence and anxieties about treatment intensification.
Study outputs
Rushforth et al. [73]

**Table 6:** Using the TDF to understand effect size; the example of post-fracture management of patients at risk of osteoporosis

Study title
Understanding effects in reviews of implementation interventions using the Theoretical Domains Framework
Rationale for changing behaviour
There is evidence that two behaviours related to post-fracture management of patients at risk of osteoporosis are sub-optimally performed: 1) primary and secondary healthcare professionals scanning bone mineral density and 2) prescribing anti-resorptive therapy (bisphosphonate medication). This study used the TDF to identify which theoretical factors were targeted in a systematic review of interventions to improve quality of care in post-fracture investigation and their relation to observed effect sizes.
Study design and materials
A behavioural scientist and a clinician independently coded TDF domains in intervention and control groups in 10 interventions identified in a systematic review. For example, part of an intervention describing an ‘algorithm for diagnosis and treatment of osteoporosis’ was coded in the domain memory, attention and decision processes. Pearson’s correlations were used to explore the relationship between intervention effect size and total number of domains identified in reviews.
Findings and conclusions
The five domains coded most frequently (in order of frequency highest to lowest) were: 1. Memory, attention and decision processes 2. Knowledge 3. Environmental context and resources 4. Social influences 5. Beliefs about consequences Correlational analysis identified a statistically significant inverse relationship between both the domain count and frequency with the observed effect size in interventions for scanning bone mineral density, i.e. interventions with a small number of domains coded infrequently tended to have larger effect sizes than interventions with a greater number of domains coded more frequently. This relationship was not observed for interventions to improve bisphosphonate prescribing.
Study outputs
Little et al. [74]

**Table 7:** Sampling for maximum variation when using TDF to understand influences on behaviour

Study title
A study of the perceived risks, benefits and barriers to the use of selective decontamination of the digestive tract (SDD) in adult critical care units
Rationale for changing behaviour
Critically ill patients who require management in an Intensive Care Unit (ICU) are particularly susceptible to hospital acquired infections which are associated with high morbidity and mortality. SDD may reduce these infections and improve mortality but has not been widely adopted into practice. Adoption of SDD would involve a set of protocolised behaviours performed by a range of healthcare professionals, so this investigation sought the views of multiple professional stakeholders.
Study design and materials
A four-phase study in three regions (the UK, Canada and Australia/New Zealand) of which Phase 2 was a Delphi study. Round 1 of the Delphi study involved one-to-one telephone interviews based on the TDF. Four key clinician groups (ICU physicians, ICU pharmacists, infectious disease clinicians/medical microbiologists, ICU clinical leads/nurse managers) were sampled using databases within each region. The researchers aimed for 10 from each group in each region. Purposive diversity sampling was used to identify a wide range of views, based on the following variables: • Hospital is academic-affiliated or not • Years of experience (time working in intensive care) • Size of ICU (number of beds) • Current practice (routinely perform SDD or not) Potential participants were ranked according to these variables and invited to participate in the Delphi study based on their ranking. During the interview phase, diversity on these factors was tracked using a diversity sampling table.
Findings and conclusions
141 participants were interviewed. Beliefs about Consequences was the most populous domain. “SDD increases antibiotic resistance”, “SDD reduces Ventilator Associated Pneumonia” and “SDD benefits the patients to whom it is delivered” were the most frequently mentioned beliefs, illustrating the problematic balance between potential harms and benefits.
Study outputs
Cuthbertson et al. [75, 76]; Dombrowski et al. [77]; Francis et al. [78]; Duncan et al. [79]; Marshall et al. [80]

**Table 8:** Reaching agreement when coding data using TDF and identifying beliefs within domains

Study title
Anaesthesiologists’ and Surgeons’ Perceptions about Routine Pre-operative testing in low risk patients: application of the Theoretical Domains Framework (TDF) to identify factors that influence physicians’ decisions to order pre-operative tests.
Rationale for changing behaviour
Routine pre-operative tests for anaesthesia management are ordered by both anaesthesiologists and surgeons for healthy patients undergoing low-risk surgery, often without any clinical indication and the subsequent test results are rarely used. Identifying factors that influence why anaesthesiologists’ and surgeons’ order these routine tests for healthy patients undergoing low risk surgery provide more effective targets for intervention development.
Study design and materials
Interview study–sixteen clinicians (eleven anaesthesiologists and five surgeons) throughout Ontario were recruited. An interview guide based on the TDF was developed to identify beliefs about pre-operative testing practices. Physicians’ statements were content analysed into the relevant theoretical domains. Two researchers coded interview participants’ statements into the relevant theoretical domains. The first pilot interview was coded in tandem to develop the coding strategy and the second was used to ensure the two coders were comfortable with the strategy developed from the first. Subsequent coding of the remaining interviews was completed independently and Fleiss’s Kappa (κ) was calculated for all domains and interviews to assess whether the two researchers coded the same text into the same domain. Within each domain, the primary coder wrote a belief statement that captured the core thought of each utterance. For example, the following utterances were coded under the domains Social Influences: “… if a surgeon ordered it I am somewhat reluctant to cancel one of their tests even though I don’t feel that it’s necessary” & “Sometimes they are ordered and then (we) might be reluctant to cancel some of the tests because I am not privy to their thought process….”. These 2 utterances were from 2 different respondents but reflect the same core thought: I’m reluctant to cancel tests ordered by other physicians. Identical beliefs statements were then grouped together. Statements that centred on same theme or were polar opposites of a theme were also grouped together for the ease of further analysis. For example, the following 3 belief statements from Social Influences grouped under the theme influence of colleagues: The opinions of others do not influence my decision to order routine tests. I’m reluctant to cancel test ordered by other physicians. I order tests I feel are unnecessary because my conservative colleague may be in the operating room on the day of the surgery and want to see the routine test that I would not. Belief statements that were coded in different domains by the researchers were discussed to establish consensus. Where single domain allocation agreement could not be reached, researchers agreed that the statement could be placed in both domains.
Findings and conclusions
Seven domains were identified as likely relevant to changing clinicians’ behaviour about pre-operative test ordering for anaesthesia management (Social/professional role and identity, Beliefs about capabilities and Social influences, Environmental context and resources, Beliefs about consequences, Behavioural regulation, Nature of the behaviour). Key beliefs identified within these domains included: conflicting comments about who was responsible for the test-ordering, inability to cancel tests ordered by fellow physicians, and the problem with tests being completed before anaesthesiologists see patients. Anaesthesiologists often ordered tests based on who may be the attending anaesthesiologist on the day of surgery while surgeons ordered tests they thought anaesthesiologists might need. There was also a range of comments about the consequences associated with reducing testing, from negative (delay or cancel patients’ surgeries), to indifference (little or no change in patient outcomes), to positive (save money, avoid unnecessary investigations).
Study outputs
Patey et al. [28]

**Table 9:** Identifying key domains to target in an intervention

Study title
A cross-country comparison of intensive care physicians’ beliefs about their transfusion behaviour: A qualitative study using the theoretical domains framework.
Rationale for changing behaviour
Transfusion of blood, a scarce and costly resource, is used for treating a variety of medical conditions. There is a wide variation in blood transfusion behaviour across different medical disciplines including intensive care physicians. A restrictive transfusion is, at least, equivalent and possibly superior to a more liberal transfusion. The aim of the study was to elicit beliefs about specified behaviour within each theoretical domain and role of the domain in influencing the behaviour in intensive care units across Canada.
Study design and materials
Ten intensive care physicians throughout Canada were interviewed. Physicians’ responses were coded into theoretical domains, and specific beliefs were generated for each response. Theoretical domains relevant to behaviour change were identified if they included belief statements that might be potential barriers for changing transfusion behaviour and fulfilled the following criteria: (1) relatively high frequency of specific beliefs, (2) presence of conflicting beliefs, and (3) evidence of strong beliefs that may impact on the behaviour. All three criteria were considered concurrently to judge relevance of the domains. Beliefs within the domains were analysed for psychological constructs and were subsequently used to select psychological theories using the methodology proposed by Francis et al. [25].
Findings and conclusions
Seven theoretical domains populated by 31 specific beliefs were identified as relevant to the target behaviour using all criteria. The relevant theoretical domains were Knowledge, Social/professional role and identity, Beliefs about capabilities, Beliefs about consequences, Motivation and goals, Social influences and Behavioural regulation. For example, Knowledge domain was identified as potentially relevant because majority participants reported the belief that there is not enough evidence to support watching and waiting in all patient populations. Motivation and goals was identified as a key domain because conflicting specific beliefs were elicited (e.g. Watching and waiting conflicts with other goals in opposition to Watching and waiting is compatible with other goals). When the belief that ‘emotion does not affect my decision to transfuse’ was consistently reported, it was concluded that the Emotion domain was not relevant to the transfusion behaviour. For greater detail please see the published article.
Study outputs
Islam et al. [26]

### Time estimates for conducting research using TDF

The time to complete each of the steps described will be highly variable according to available resources such as staff, funding and any restrictions on timing. We provide broad estimates of days, weeks or months to complete the steps:

- Steps 1–3: Selecting and specifying the target behaviour, selecting study design, and deciding the sampling strategy may take days or weeks. In relation to identification of the target behaviour, conducting interviews and follow-up work will have cost implications so there needs to be good evidence that changing the behaviour in question will produce benefits and reduce harms.
- Step 4: Developing study materials may take weeks to months to produce, pilot and finalise interview schedules and topic guides for focus groups.
- Step 5: Collecting data is likely to take months to complete. In some cases, it may take 1 month to complete interviews but it can easily take several times longer depending on the numbers required and difficulties with recruitment.
- Step 6: Analysing the data may take months and will depend on the amount of data, number of staff coding the data and number of disagreements in coding the data.

In summary, a TDF-based interview study can take around 12 months to complete. We are aware of groups completing studies in a few months and others taking up to 24 months. Time to complete will vary according to the size and scope of the study, demands of ethics requirements, extent of rigour, i.e. whether conducted for local purposes or for publication, existing expertise and dedicated research staff.

### Report findings

Findings of TDF-based interview studies are reported in tables as well as text to provide a rich and clear description of the influences on the implementation problem. Tables include quotations from transcripts, summary statements generated from these quotations, frequency counts and/or emerging themes depending on methodology used. A good example of tabulating data gathered using TDF is provided in Patey et al. [28].

## Discussion

This step-by-step guide for applying the Theoretical Domains Framework of behaviour change to implementation problems using qualitative data approaches has been developed as a resource for the implementation research community. The benefits of using the TDF are that it provides a robust theoretical basis for implementation studies, good coverage of potential reasons for implementation problems and, in conjunction with other tools and methods, a methodology for progressing from investigation to intervention. It is clear from the volume of research in this field, especially in the exploratory stages of multidisciplinary research programmes, that the TDF has opened up new approaches to investigating and addressing problems of implementation. As methods and programmes mature, more evidence will become available from which to assess the added value of using the TDF to inform both intervention design and future version of this guide.

### Potential applications of the TDF

The TDF may be used to guide data collection using, for example, interviews, focus groups, structured observation and questionnaires designed to identify barriers and facilitators to change. It can also be used in predictive studies to examine the relationships between theoretical domains and uptake of a target behaviour and process evaluations to identify mechanisms of change. It can be used to synthesise evidence in systematic reviews of literature and to guide behaviour change technique selection when designing interventions. Whilst the framework has been used primarily in healthcare settings for exploring factors influencing clinical behaviours to design implementation interventions, it is also relevant for designing interventions related to population or public health, occupational health as well as non-health behaviours, e.g. environmental, transport related.

### Linking TDF to other theoretical models

**Fig. 2:**
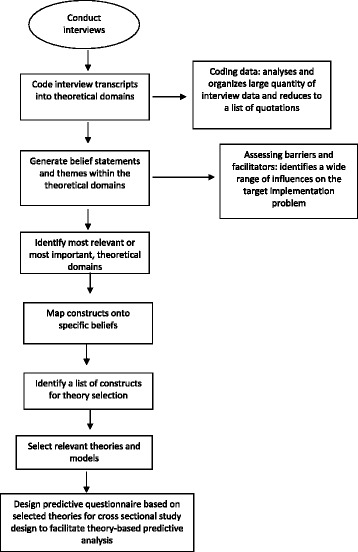
Linking TDF to the COM-B model [43]

The TDF has been linked to a more recently developed, simpler model of behaviour, the COM-B model [16, 43]. The central tenet of this model is that capability, opportunity and motivation interact to produce behaviour. TDF provides a more granular understanding of psychological capability and reflective motivational processes (see Fig. 2). The example in Table 10 illustrates how linking COM-B and TDF was helpful when researchers had limited time with participants to conduct focus group interviews (also reported in Michie et al. [43]). Domains of the TDF can also direct researchers to other relevant theories and frameworks. For example, the social influences and environmental context and resource domains point to organisational and systems context for change and can be further elaborated, e.g. by Normalisation Process Theory [66], the Consolidated Framework For Implementation Research [67] and the Yorkshire Contributory Factors Framework [68].

**Table 10:** Linking TDF to a theoretical model to maximise coverage of domains under time constraints

Study title
Factors Influencing Variation in Physician Adenoma Detection Rates: a Theory-Based Approach for Performance Improvement.
Rationale for changing behaviour
Interventions to improve physician adenoma detection rates (ADRs) for colonoscopy have generally not been successful. There is limited understanding of which factors influence variation which might be appropriate targets for intervention.
Study design and materials
Three focus groups of gastroenterologists and three of endoscopy nurses were conducted at medical centres in Northern California. As participants were available for a limited time (45–60 minutes), an adaptive interviewing method was used. First, participants were asked questions covering the three components of the COM-B model (capability, opportunity and motivation) to identify factors relevant in explaining ADR variation. Then for each relevant COM-B component, participants were asked questions covering the related domains of the TDF. For example, to investigate participants’ capabilities to perform a behaviour, they were asked “would you be more/less likely to do ‘X’ if you had greater physical and/or psychological ability?” If they responded positively, the researcher asked further questions structured by TDF domains representing capability, i.e. knowledge; physical skills; memory, attention and decision processes and behavioural regulation.
Findings and conclusions
This adaptive interviewing method optimised the time available with higher level COM-B questions acting as a filter to potentially relevant TDF domains.
Study outputs
Atkins et al. [81]

### Limitations

There are two key limitations to this guide. First, the scope is mostly limited to qualitative approaches to using the TDF, mainly interview and focus group data, and provides limited detail on using the TDF with other data collection methods such as survey or observation. Secondly, whilst the group selected illustrative applications of the TDF from the peer-reviewed literature, this was based upon an informal rather than structured consensus process leaving the possibility of selection bias in these examples. However, the aim was not to be representative but to provide examples of the application of the TDF in a range of settings for a range of implementation problems.

### Directions for future research

Whilst there is evidence of the TDF being used to investigate fidelity of intervention delivery, we did not identify any examples in the literature investigating either fidelity of TDF application or harms or unintended consequences of using the TDF. To ensure optimal application of the TDF, we suggested these as possible areas for future methodological research.

## Conclusions

This guide is a response to calls for more explicit guidance on applying the TDF to understand implementation problems [21]. To our knowledge, this is the first of its kind. We envisage future versions of this guide as methods and evidence in the field of *Implementation Science* moves forward.

## Additional files

Additional file 1: Published examples of TDF applications [7, 20, 25, 26, 28, 30, 36, 41, 42, 48, 49, 73, 76, 78, 79, 82, 83]. (DOCX 35 kb)

Additional file 2: Example questions to explore domains in implementation research. Taken from Huijg et al. [49]. (DOCX 16 kb)

Additional file 3: Extracts from interview transcripts coded using the TDF [81]. (DOCX 844 kb)

## References

[1] Eccles M, Grimshaw J, Walker A, Johnston M, Pitts N (2005). Changing the behavior of healthcare professionals: the use of theory in promoting the uptake of research findings. J Clin Epidemiol.

[2] Foy R, Eccles MP, Jamtvedt G, Young J, Grimshaw JM, Baker R (2005). What do we know about how to do audit and feedback? Pitfalls in applying evidence from a systematic review. BMC Health Serv Res.

[3] Dixon-Woods M, Bosk CL, Aveling EL, Goeschel CA, Pronovost PJ (2011). Explaining Michigan: developing an ex post theory of a quality improvement program. Milbank Q.

[4] Eccles MP, Grimshaw JM, MacLennan G, Bonetti D, Glidewell L, Pitts NB (2012). Explaining clinical behaviors using multiple theoretical models. Implement Sci.

[5] Michie S (2008). Designing and implementing behaviour change interventions to improve population health. J Health Serv Res Policy.

[6] The Improved Clinical Effectiveness through Behavioural Research Group (ICEBeRG) (2006). Designing theoretically-informed implementation interventions. Implement Sci.

[7] Michie S, Johnston M, Francis J, Hardeman W, Eccles M (2008). From theory to intervention: mapping theoretically derived behavioural determinants to behaviour change techniques. Appl Psychol.

[8] Davies P, Walker AE, Grimshaw JM (2010). A systematic review of the use of theory in the design of guideline dissemination and implementation strategies and interpretation of the results of rigorous evaluations. Implement Sci.

[9] Craig P, Dieppe P, Macintyre S, Michie S, Nazareth I, Petticrew M (2008). Developing and evaluating complex interventions: the new Medical Research Council guidance. BMJ.

[10] Foy R, Ovretveit J, Shekelle PG, Pronovost PJ, Taylor SL, Dy S (2011). The role of theory in research to develop and evaluate the implementation of patient safety practices. BMJ Qual Saf.

[11] Michie S, Prestwich A (2010). Are interventions theory-based? Development of a theory coding scheme. Health Psychol.

[12] Michie S, Webb TL, Sniehotta FF (2010). The importance of making explicit links between theoretical constructs and behaviour change techniques. Addiction.

[13] Baker R, Camosso-Stefinovic J, Gillies C, Shaw EJ, Cheater F, Flottorp S, et al. Tailored interventions to address determinants of practice. Cochrane Database of Systematic Reviews. 2015;4:CD005470. doi:10.1002/14651858.CD005470.pub3. 10.1002/14651858.CD005470.pub3 PMC7271646 25923419

[14] Colquhoun HL, Brehaut JC, Sales A, Ivers N, Grimshaw J, Michie S (2013). A systematic review of the use of theory in randomized controlled trials of audit and feedback. Implement Sci.

[15] Michie S, Johnston M, Abraham C, Lawton R, Parker D, Walker A (2005). Making psychological theory useful for implementing evidence based practice: a consensus approach. Qual Saf Health Care.

[16] Cane J, O’Connor D, Michie S (2012). Validation of the theoretical domains framework for use in behaviour change and implementation research. Implement Sci.

[17] Kolehmainen N, Francis JJ, Ramsay CR, Owen C, McKee L, Ketelaar M (2011). Participation in physical play and leisure: developing a theory- and evidence-based intervention for children with motor impairments. BMC Pediatr.

[18] Nicholson SL, Donaghy M, Johnston M, Sniehotta FF, van Wijck F, Johnston D (2014). A qualitative theory guided analysis of stroke survivors’ perceived barriers and facilitators to physical activity. Disabil Rehabil.

[19] Honigh-de Vlaming R, Haveman-Nies A, Heinrich J, van’t Veer P, de Groot LC (2013). Effect evaluation of a two-year complex intervention to reduce loneliness in non-institutionalised elderly Dutch people. BMC Public Health.

[20] Taylor N, Lawton R, Conner M (2013). Development and initial validation of the determinants of physical activity questionnaire. Int J Behav Nutr Phys Act.

[21] Phillips CJ, Marshall AP, Chaves NJ, Jankelowitz SK, Lin IB, Loy CT (2015). Experiences of using the Theoretical Domains Framework across diverse clinical environments: a qualitative study. J Multidiscip Healthc.

[22] Davis R, Campbell R, Hildon Z, Hobbs L, Michie S (2015). Theories of behaviour and behaviour change across the social and behavioural sciences: a scoping review. Health Psychol Rev.

[23] Francis JJ, O’Connor D, Curran J. Theories of behaviour change synthesised into a set of theoretical groupings: introducing a thematic series on the theoretical domains framework. Implement Sci. 2012;7:35. https://implementationscience.biomedcentral.com/articles/10.1186/1748-5908-7-35. 10.1186/1748-5908-7-35 PMC3444902 22531601

[24] Michie S, Pilling S, Garety P, Whitty P, Eccles MP, Johnston M (2007). Difficulties implementing a mental health guideline: an exploratory investigation using psychological theory. Implement Sci.

[25] Francis JJ, Stockton C, Eccles MP, Johnston M, Cuthbertson BH, Grimshaw JM, et al. Evidence-based selection of theories for designing behaviour change interventions: using methods based on theoretical construct domains to understand clinicians’ blood transfusion behaviour. Br J Health Psychol. 2009;14(Pt 4):625–46. 10.1348/135910708X397025 19159506

[26] Islam R, Tinmouth AT, Francis JJ, Brehaut JC, Born J, Stockton C (2012). A cross-country comparison of intensive care physicians’ beliefs about their transfusion behaviour: a qualitative study using the Theoretical Domains Framework. Implement Sci.

[27] McSherry LA, Dombrowski SU, Francis JJ, Murphy J, Martin CM, O’Leary JJ (2012). ‘It’s a can of worms’: understanding primary care practitioners’ behaviours in relation to HPV using the theoretical domains framework. Implement Sci.

[28] Patey AM, Islam R, Francis JJ, Bryson GL, Grimshaw JM, Canada PPT (2012). Anesthesiologists’ and surgeons’ perceptions about routine pre-operative testing in low-risk patients: application of the Theoretical Domains Framework (TDF) to identify factors that influence physicians’ decisions to order pre-operative tests. Implement Sci.

[29] Duncan EM, Francis JJ, Johnston M, Davey P, Maxwell S, McKay GA (2012). Learning curves, taking instructions, and patient safety: using a theoretical domains framework in an interview study to investigate prescribing errors among trainee doctors. Implement Sci.

[30] Bussieres AE, Patey AM, Francis JJ, Sales AE, Grimshaw JM, Canada PPT (2012). Identifying factors likely to influence compliance with diagnostic imaging guideline recommendations for spine disorders among chiropractors in North America: a focus group study using the Theoretical Domains Framework. Implement Sci.

[31] Murphy K, O’Connor DA, Browning CJ, French SD, Michie S, Francis JJ (2014). Understanding diagnosis and management of dementia and guideline implementation in general practice: a qualitative study using the theoretical domains framework. Implement Sci.

[32] Tavender EJ, Bosch M, Gruen RL, Green SE, Knott J, Francis JJ (2014). Understanding practice: the factors that influence management of mild traumatic brain injury in the emergency department—a qualitative study using the Theoretical Domains Framework. Implement Sci.

[33] Dyson J, Lawton R, Jackson C, Cheater F (2011). Does the use of a theoretical approach tell us more about hand hygiene behaviour? The barriers and levers to hand hygiene. J Infect Prev.

[34] Amemori M, Michie S, Korhonen T, Murtomaa H, Kinnunen TH (2011). Assessing implementation difficulties in tobacco use prevention and cessation counselling among dental providers. Implement Sci.

[35] Beenstock J, Sniehotta FF, White M, Bell R, Milne EM, Araujo-Soares V (2012). What helps and hinders midwives in engaging with pregnant women about stopping smoking? A cross-sectional survey of perceived implementation difficulties among midwives in the North East of England. Implement Sci.

[36] French SD, Green SE, O’Connor DA, McKenzie JE, Francis JJ, Michie S (2012). Developing theory-informed behaviour change interventions to implement evidence into practice: a systematic approach using the Theoretical Domains Framework. Implement Sci.

[37] McKenzie JE, O’Connor DA, Page MJ, Mortimer DS, French SD, Walker BF (2010). Improving the care for people with acute low-back pain by allied health professionals (the ALIGN trial): a cluster randomised trial protocol. Implement Sci.

[38] Tavender EJ, Bosch M, Gruen RL, Green SE, Michie S, Brennan SE (2015). Developing a targeted, theory-informed implementation intervention using two theoretical frameworks to address health professional and organisational factors: a case study to improve the management of mild traumatic brain injury in the emergency department. Implement Sci.

[39] Backman R, Foy R, Michael BD, Defres S, Kneen R, Solomon T (2015). The development of an intervention to promote adherence to national guidelines for suspected viral encephalitis. Implement Sci.

[40] Taylor N, Lawton R, Moore S, Craig J, Slater B, Cracknell A (2014). Collaborating with front-line healthcare professionals: the clinical and cost effectiveness of a theory based approach to the implementation of a national guideline. BMC Health Serv Res.

[41] Curran JA, Brehaut J, Patey AM, Osmond M, Stiell I, Grimshaw JM. Understanding the Canadian adult CT head rule trial: use of the theoretical domains framework for process evaluation. Implement Sci. 2013;8. doi:10.1186/1748-5908-8-25. 10.1186/1748-5908-8-25 PMC3585785 23433082

[42] Cane J, Richardson M, Johnston M, Ladha R, Michie S (2015). From lists of behaviour change techniques (BCTs) to structured hierarchies: comparison of two methods of developing a hierarchy of BCTs. Br J Health Psychol.

[43] Michie S, Atkins L, West R (2014). The Behaviour Change Wheel—a guide to designing interventions.

[44] Gainforth H, Sheals K, Atkins L, Jackson R, Michie S (2016). Developing interventions to change recycling behaviors: a case study of applying behavioral science. J Appl Environ Educ Commun.

[45] Presseau J, Francis JJ, Campbell NC, Sniehotta FF (2011). Goal conflict, goal facilitation, and health professionals’ provision of physical activity advice in primary care: an exploratory prospective study. Implement Sci.

[46] Fishbein M (1967). Readings in attitude theory and measurement.

[47] Francis J, Eccles MP, Johnston M, Walker AE, Grimshaw JM, Foy R, Kaner EFS (2004). Constructing questionnaires based on the theory of planned behaviour: a manual for health services researchers.

[48] Taylor N, Parveen S, Robins V, Slater B, Lawton R. Development and initial validation of the Influences on Patient Safety Behaviours Questionnaire. Implement Sci. 2013;8. doi:10.1186/1748-5908-8-81. 10.1186/1748-5908-8-81 PMC3846501 23895628

[49] Huijg JM, Gebhardt WA, Crone MR, Dusseldorp E, Presseau J. Discriminant content validity of a theoretical domains framework questionnaire for use in implementation research. Implement Sci. 2014;9. doi:10.1186/1748-5908-9-11. 10.1186/1748-5908-9-11 PMC3896680 24423394

[50] Ferlie EB, Shortell SM (2001). Improving the quality of health care in the United Kingdom and the United States: a framework for change. Milbank Q.

[51] Miller DT, Ross M (1975). Self-serving biases in the attribution of causality: fact or fiction. Psychol Bull.

[52] Zuckerman M (1979). Attribution of success and failure revisited, or: the motivational bias is alive and well in attribution theory. J Pers.

[53] Squires JE, Suh KN, Linklater S, Bruce N, Gartke K, Graham ID, et al. Improving physician hand hygiene compliance using behavioural theories: a study protocol. Implement Sci. 2013;8. doi:10.1186/1748-5908-8-16. 10.1186/1748-5908-8-16 PMC3571966 23379466

[54] Steinmo S, Fuller C, Stone SP, Michie S (2015). Characterising an implementation intervention in terms of behaviour change techniques and theory: the ‘Sepsis Six’ clinical care bundle. Implement Sci.

[55] O’Cathain A, Murphy E, Nicholl J (2010). Three techniques for integrating data in mixed methods studies. BMJ.

[56] Francis JJ, Johnston M, Robertson C, Glidewell L, Entwistle V, Eccles MP (2010). What is an adequate sample size? Operationalising data saturation for theory-based interview studies. Psychol Health.

[57] Hsieh HF, Shannon SE (2005). Three approaches to qualitative content analysis. Qual Health Res.

[58] Glaser BG, Strauss AL (1967). The discovery of grounded theory: strategies for qualitative research.

[59] Charmaz K (2006). Constructing grounded theory: a practical guide through qualitative analysis.

[60] Ritchie J, Spencer L, O'Connor W, Ritchie J, Lewis J (2003). Carrying out qualitative analysis. Qualitative research practice: a guide for social science students and researchers.

[61] Morse J (2000). Determining sample size. Qual Health Res.

[62] Birkimer JC, Brown JH (1979). Back to basics: percentage agreement measures are adequate, but there are easier ways. J Appl Behav Anal.

[63] Landis JR, Koch GG (1977). The measurement of observer agreement for categorical data. Biometrics.

[64] Braun V, Clarke V (2006). Using thematic analysis in psychology. Qual Res Psychol.

[65] Marks DF and Yardley L. Research methods for clinical and health psychology. London: Sage; 2004.

[66] May CR, Mair F, Finch T, MacFarlane A, Dowrick C, Treweek S (2009). Development of a theory of implementation and integration: Normalization Process Theory. Implement Sci.

[67] Damschroder LJ, Aron DC, Keith RE, Kirsh SR, Alexander JA, Lowery JC (2009). Fostering implementation of health services research findings into practice: a consolidated framework for advancing implementation science. Implement Sci.

[68] Lawton R, McEachan RR, Giles SJ, Sirriyeh R, Watt IS, Wright J (2012). Development of an evidence-based framework of factors contributing to patient safety incidents in hospital settings: a systematic review. BMJ Qual Saf.

[69] French SD, McKenzie JE, O’Connor DA, Grimshaw JM, Mortimer D, Francis JJ, et al. Evaluation of a theory-informed implementation intervention for the management of acute low back pain in general medical practice: the IMPLEMENT Cluster Randomised Trial. PloS One. 2013;8(6):e65471. doi:10.1371/journal.pone.0065471. 10.1371/journal.pone.0065471 PMC3681882 23785427

[70] Page MJ, French SD, McKenzie JE, O’Connor DA, Green SE (2011). Recruitment difficulties in a primary care cluster randomised trial: investigating factors contributing to general practitioners’ recruitment of patients. BMC Med Res Methodol.

[71] McKenzie JE, French SD, O’Connor DA, Grimshaw JM, Mortimer D, Michie S (2008). IMPLEmenting a clinical practice guideline for acute low back pain evidence-based manageMENT in general practice (IMPLEMENT): cluster randomised controlled trial study protocol. Implement Sci.

[72] Taylor N, Lawton R, Slater B, Foy R. The demonstration of a theory-based approach to the design of localized patient safety interventions. Implement Sci. 2013;8. doi:10.1186/1748-5908-8-123. 10.1186/1748-5908-8-123 PMC3854455 24131864

[73] Rushforth B, McCrorie C, Glidewell L, Midgley E, Foy R (2016). Barriers to effective management of type 2 diabetes in primary care: qualitative systematic review. Br J Gen Pract.

[74] Little EA, Presseau J, Eccles MP (2015). Understanding effects in reviews of implementation interventions using the Theoretical Domains Framework. Implement Sci.

[75] Cuthbertson BH, Campbell MK, MacLennan G, Duncan EM, Marshall AP, Wells EC, et al. Clinical stakeholders’ opinions on the use of selective decontamination of the digestive tract in critically ill patients in intensive care units: an international Delphi study. Crit Care. 2013;17(6):R266. doi:10.1186/cc13096. 10.1186/cc13096 PMC4056354 24207137

[76] Cuthbertson BH, Francis J, Campbell MK, MacIntyre L, Seppelt I, Grimshaw J, et al. A study of the perceived risks, benefits and barriers to the use of SDD in adult critical care units (The SuDDICU study). Trials. 2010;11:117. doi:10.1186/1745-6215-11-117. 10.1186/1745-6215-11-117 PMC3017022 21129208

[77] Dombrowski SU, Prior ME, Duncan E, Cuthbertson BH, Bellingan G, Campbell MK (2013). Clinical components and associated behavioural aspects of a complex healthcare intervention: multi-methods study of selective decontamination of the digestive tract in critical care. Aust Crit Care.

[78] Francis JJ, Duncan EM, Prior ME, MacLennan GS, Dombrowski SU, Bellingan G (2014). Selective decontamination of the digestive tract in critically ill patients treated in intensive care units: a mixed-methods feasibility study (the SuDDICU study). Health Technol Assess.

[79] Duncan EM, Cuthbertson BH, Prior ME, Marshall AP, Wells EC, Todd LE (2014). The views of health care professionals about selective decontamination of the digestive tract: an international, theoretically informed interview study. J Crit Care.

[80] Marshall AP, Weisbrodt L, Rose L, Duncan E, Prior M, Todd L (2014). Implementing selective digestive tract decontamination in the intensive care unit: a qualitative analysis of nurse-identified considerations. Heart Lung.

[81] Atkins L, Hunkeler EM, Jensen CD, Michie S, Lee JK, Doubeni CA (2016). Factors influencing variation in physician adenoma detection rates: a theory-based approach for performance improvement. Gastrointest Endosc.

[82] Squires JE, Linklater S, Grimshaw JM, Graham ID, Sullivan K, Bruce N (2014). Understanding practice: factors that influence physician hand hygiene compliance. Infect Control Hosp Epidemiol.

[83] Heslehurst N, Newham J, Maniatopoulos G, Fleetwood C, Robalino S, Rankin J (2014). Implementation of pregnancy weight management and obesity guidelines: a meta-synthesis of healthcare professionals’ barriers and facilitators using the Theoretical Domains Framework. Obes Rev.

