# Supplementary material for: A guide to using the Theoretical Domains Framework of behaviour change to investigate implementation problems
Source: Implement Sci. 2017 Jun 21;12:77. doi: 10.1186/s13012-017-0605-9 (PMC5480145; doi:10.1186/s13012-017-0605-9)
Supplement: Supplementary file 1 — Published examples of TDF applications [7, 20, 25, 26, 28, 30, 36, 41, 42, 48, 49, 73, 76, 78, 79, 82, 83]. (DOCX 35 kb) [file 13012_2017_605_MOESM1_ESM.docx]

**Additional file 1. Published examples of TDF applications**

| **Study objective** | **Key publications** |
| --- | --- |
| Explore implementation problems | 1. Francis JJ, Stockton C, Eccles MP, Johnston M, Cuthbertson BH, Grimshaw JM et al. Evidence-based selection of theories for designing behaviour change interventions: Using methods based on theoretical construct domains to understand clinicians' blood transfusion behaviour. British Journal of Health Psychology. 2009;14:625-46. [25] 2. Islam R, Tinmouth AT, Francis JJ, Brehaut JC, Born J, Stockton C et al. A cross-country comparison of intensive care physicians' beliefs about their transfusion behaviour: a qualitative study using the theoretical domains framework. Implementation Science. 2012;7:93. [26] 3. Patey AM, Islam R, Francis JJ, Bryson GL, Grimshaw JM. Anesthesiologists and surgeons’ perceptions about routine pre-operative testing in low-risk patients: application of the Theoretical Domains Framework (TDF) to identify factors that influence physicians decisions to order pre-operative tests. Implementation Science. 2012;7:52. [28] 4. Bussieres AE, Patey AM, Francis JJ, Sales AE, Jeremy GM, Canada PRIme Plus Team. Identifying factors likely to influence compliance with diagnostic imaging guideline recommendations for spine disorders among chiropractors in North America: a focus group study using the Theoretical Domains Framework. Implementation Science. 2012;7:82. [30] 5. Cuthbertson BH, Francis J, Campbell M, MacIntyre L, Sepelt I, Grimshaw J. A study of the perceived risks, benefits and barriers to the use of SDD in adult critical care units. Trials. 2010;11:117. [76] 6. Duncan EM, Cuthbertson BH, Prior ME, Marshall AP, Wells EC, Todd, LE et al.   The views of health care professionals about Selective Decontamination of the Digestive tract: an international, theoretically-informed study. Journal of Critical Care. 2014;29(4):634-40. [79]   1. Francis JJ, Duncan EM, Prior ME, MacLennan GS, Dombrowski SU, Bellingan G et al. Selective decontamination of the digestive tract in critically ill patients treated in Intensive Care Units: a mixed-methods feasibility study. Health Technology Assessment. 2014;18(25). [78] 2. Squires J, Linklater S, Grimshaw JM, Graham ID, Sullivan K, Bruce N et al. Understanding Practice: Factors That Influence Physician Hand Hygiene Compliance. Infection Control and Hospital Epidemiology. 2014;35(12):1511-1520. [82] |
| Systematic selection of psychological theories | 1. Francis JJ, Stockton C, Eccles MP, Johnston M, Cuthbertson BH, Grimshaw JM et al. Evidence-based selection of theories for designing behaviour change interventions: Using methods based on theoretical construct domains to understand clinicians' blood transfusion behaviour. British Journal of Health Psychology. 2009;14:625-46. [25] 2. Islam R, Tinmouth AT, Francis JJ, Brehaut JC, Born J, Stockton C et al. A cross-country comparison of intensive care physicians' beliefs about their transfusion behaviour: a qualitative study using the theoretical domains framework. Implementation Science. 2012;7:93. [26] 3. Patey AM, Islam R, Francis JJ, Bryson GL, Grimshaw JM. Anesthesiologists and surgeons’ perceptions about routine pre-operative testing in low-risk patients: application of the Theoretical Domains Framework (TDF) to identify factors that influence physicians decisions to order pre-operative tests. Implementation Science. 2012;7:52. [28] 4. Bussieres AE, Patey AM, Francis JJ, Sales AE, Jeremy GM, Canada PRIme Plus Team. Identifying factors likely to influence compliance with diagnostic imaging guideline recommendations for spine disorders among chiropractors in North America: a focus group study using the Theoretical Domains Framework. Implementation Science. 2012;7:82. [30] |
| Theory-based intervention development | 1. French SD, Green SE, O’Connor DA, McKenzie JE, Francis JJ, Michie S et al. Developing theory-informed behaviour change interventions to implement evidence into practice: a systematic approach using the Theoretical Domains Framework. Implementation Science. 2012;7:38. [36] 2. Michie S, Johnston M, Francis J, Hardeman W and Eccles M. From Theory to Intervention: Mapping Theoretically Derived Behavioural Determinants to Behaviour Change Techniques. Applied Psychology: an international review. 2008;57(4):660-680. [7] 3. Cane J, Richardson M, Johnston M, Ladha R, Michie S. From lists of behaviour change techniques (BCTs) to structured hierarchies: Comparison of two methods of developing a hierarchy of BCTs. British Journal of Health Psychology. 2015;20(1):130-150. [42] |
| Systematic review | 1. Rushforth B, McCrorie C, Glidewell L, Midgley E, Foy R. Barriers to effective management of type 2 diabetes in primary care: Qualitative systematic review. British Journal of General Practice. 2016;66(643):e114-e127. [73] 2. Heslehurst N, Newham J, Maniatopoulos G, Fleetwood C, Robalino S, Rankin J. Implementation of pregnancy weight management and obesity guidelines: a meta-synthesis of healthcare professionals’ barriers and facilitators using the Theoretical Domains Framework. Obesity Reviews. 2014;15(6):462–486. [83] |
| Process evaluation | 1. Curran JA, Brehaut J, Patey AM, Osmond M, Stiell I, Grimshaw JM et al. Understanding the Canadian adult CT head rule trial: use of the theoretical domains framework for process evaluation. Implementation Science. 2013;8:25. [41] |
| Questionnaire design | 1. Huijg JM, Gebhardt WA, Crone ME, Dusseldorp E, Presseau J. Discriminant content validity of a theoretical domains framework questionnaire for use in implementation research. Implementation Science. 2014;9:11. [49] 2. Taylor N, Parveen S, Robins V, Slater B, Lawton R. Development and initial validation of the Influences on Patient Safety Behaviours Questionnaire. Implementation Science. 2013;8:81. [48] 3. Taylor N, Lawton R, Conner M. Development and initial validation of the determinants of physical activity questionnaire. International Journal of Behavioral Nutrition and Physical Activity. 2013;10:74. [20] |
