# Supplementary material for: A guide to using the Theoretical Domains Framework of behaviour change to investigate implementation problems
Source: Implement Sci. 2017 Jun 21;12:77. doi: 10.1186/s13012-017-0605-9 (PMC5480145; doi:10.1186/s13012-017-0605-9)
Supplement: Supplementary file 2 — Example questions to explore domains in implementation research. Taken from Huijg et al. [49]. (DOCX 16 kb) [file 13012_2017_605_MOESM2_ESM.docx]

**Additional file 2. Example questions to explore domains in implementation research. Taken from Huijg et al. [49]**

| **Domain** | **Item** |
| --- | --- |
| Knowledge | I am aware of the content and objectives of [innovation/guideline] |
|  | I know the content and objectives of [innovation/guideline] |
|  | I am familiar with the content and objectives of [innovation/guideline] |
|  | I am aware of how to [A] in [C, T] with [Ta] |
| Skills | I have been trained how to [A] in [C, T] with [Ta] |
|  | I have the skills to [A] in [C, T] with [Ta] |
|  | I have practiced [A] in [C, T] with [Ta] |
| Social/professional role and identity | [A] in [C, T] with [Ta] is part of my work as a [profession] |
|  | As a [profession], it is my job to [A] in [C, T] with [Ta] |
|  | It is my responsibility as a [profession] to [A] in [C, T] with [Ta] |
|  | Doing [A] in [C, T] with [Ta] is consistent with my [profession] |
| Beliefs about capabilities | I am confident that I can [A] in [C, T] with [Ta] even when [Ta] is not motivated |
|  | I am confident that I can [A] in [C, T] with [Ta] even when there is little time |
|  | I am confident that if I wanted I could [A] in [C, T] with [Ta] |
| Optimism | With regard to [A] in [C, T] with [Ta] in uncertain times, I usually expect the best |
|  | With regard to [A] in [C, time] with [Ta] I’m always optimistic about the future |
| Beliefs about consequences | If I [A] in [C, T] with [Ta] it will benefit public health |
|  | If I [A] in [C, T] with [Ta] it will have disadvantages for my relationship with [Ta] |
| Reinforcement* | Whenever I [A] in [C, T] with [Ta], I get financial reimbursement |
|  | Whenever I [A] in [C, T] with [Ta], I get recognition from professionals who are important to me |
| Intentions | For how many of the next 10 [Ta] do you intend to [A] in [C]? |
|  | I will definitely [A] in [C] with [Ta] in the next [T] |
|  | I intend to [A] in [C] with [Ta] in the next [T] |
|  | How strong is your intention to [A] with [Ta] in [C] in the next [T]? |
| Goals* | I have a clear plan how often I will [A] in [C, T] with [Ta] |
|  | Generally, in [C, T] with [Ta], how often is covering something else on your agenda a higher priority than [A] |
| Memory, attention and decision processes | How often do you forget [A] in [C, T] with [Ta]? |
|  | When I need to concentrate to [A] in [C, T] with [Ta], I have no trouble focusing my attention |
|  | When trying to focus my attention on [A] in [C, T] with [Ta], I have difficulty blocking out distracting thoughts |
|  | When concentrating on [A] in [C, T] with [Ta], I can focus my attention so that I become unaware of what’s going on around me |
| Environmental context and resources | Within the socio-political context there is sufficient financial support (e.g., from local authorities, insurance companies, the government) for [innovation/guideline] |
|  | Within the socio-political context there are good networks between parties involved in [innovation/guideline] |
| Social influences | Most people who are important to me think that I should [A] in [C, T] with [Ta] |
|  | Most people whose opinion I value would approve me of [A] in [C, T] with [Ta] |
| Emotion | Have you recently, during the past two weeks been able to enjoy your normal day-to-day activities? |
|  | Have you recently, during the past two weeks been feeling unhappy and depressed? |
| Behavioral regulation* | I keep track of my overall progress towards [A] in [C, T] with [Ta] |
|  | I am aware of my day-to-day behavior as I work towards [A] in [C, T] with [Ta] |

[A], action; [C], context; [T], time; [Ta], target;

* Items representing these domains did not demonstrate discriminant content validity in Huijg et al.[49]
