# Supplementary material for: A guide to using the Theoretical Domains Framework of behaviour change to investigate implementation problems
Source: Implement Sci. 2017 Jun 21;12:77. doi: 10.1186/s13012-017-0605-9 (PMC5480145; doi:10.1186/s13012-017-0605-9)
Supplement: Supplementary file 3 — Extracts from interview transcripts coded using the TDF [81]. (DOCX 844 kb) [file 13012_2017_605_MOESM3_ESM.docx]

**Additional file 3. Extracts from interview transcripts coded using the TDF [81]**

The following are extracts from focus groups with gastroenterologists to identify barriers and facilitators to performing the target behaviour of performing a colonoscopy. The interview text has been coding using TDF to reflect the domain, e.g. environmental context and resources, and the theme within the domain, e.g. time needed to perform a careful colonoscopy.

**
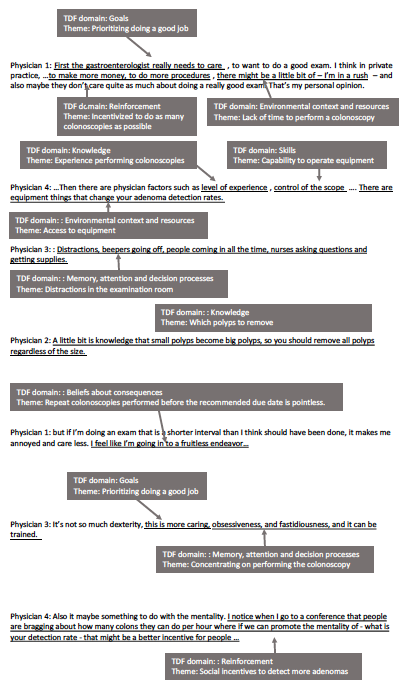
**
